# Proton beam radiation induces DNA damage and cell apoptosis in glioma stem cells through reactive oxygen species

**DOI:** 10.1038/srep13961

**Published:** 2015-09-10

**Authors:** R. Alan Mitteer, Yanling Wang, Jennifer Shah, Sherika Gordon, Marcus Fager, Param-Puneet Butter, Hyun Jun Kim, Consuelo Guardiola-Salmeron, Alejandro Carabe-Fernandez, Yi Fan

**Affiliations:** 1 Department of Radiation Oncology, University of Pennsylvania Perelman School of Medicine, Philadelphia, Pennsylvania, USA 19104; 2 Lehigh University, Bethlehem, Pennsylvania, USA 18015; 3 Drexel University, Philadelphia, Pennsylvania, USA 19104; 4 Department of Physics & Astronomy, University of Pennsylvania, Philadelphia, Pennsylvania, USA 19104

## Abstract

Glioblastoma multiforme (GBM) is among the most lethal of human malignancies. Most GBM tumors are refractory to cytotoxic therapies. Glioma stem cells (GSCs) significantly contribute to GBM progression and post-treatment tumor relapse, therefore serving as a key therapeutic target; however, GSCs are resistant to conventional radiation therapy. Proton therapy is one of the newer cancer treatment modalities and its effects on GSCs function remain unclear. Here, by utilizing patient-derived GSCs, we show that proton radiation generates greater cytotoxicity in GSCs than x-ray photon radiation. Compared with photon radiation, proton beam irradiation induces more single and double strand DNA breaks, less H2AX phosphorylation, increased Chk2 phosphorylation, and reduced cell cycle recovery from G2 arrest, leading to caspase-3 activation, PARP cleavage, and cell apoptosis. Furthermore, proton radiation generates a large quantity of reactive oxygen species (ROS), which is required for DNA damage, cell cycle redistribution, apoptosis, and cytotoxicity. Together, these findings indicate that proton radiation has a higher efficacy in treating GSCs than photon radiation. Our data reveal a ROS-dependent mechanism by which proton radiation induces DNA damage and cell apoptosis in GSCs. Thus, proton therapy may be more efficient than conventional x-ray photon therapy for eliminating GSCs in GBM patients.

Glioblastoma multiforme (GBM), the grade IV glioma, is the most common primary brain tumor in humans. GBM is among the most aggressive cancers with a median survival of approximately 14 months, largely due to GBM being resistant to current radio- and chemotherapies^1^,^2^. Recent studies have identified a prominent population of glioma stem cells (GSCs), *i.e.*, tumor-initiating cells or tumor-propagating cells in GBM, which are pluripotent and have the ability to repopulate tumors^3^,^4^. Most GSCs are radio-resistant and responsible for tumor recurrence^4^. Therefore, the development of therapies that are efficient at eradicating GSCs is crucial for GBM treatment moving forward.

Proton therapy is one of the newer radiation treatment modalities and when compared with conventional x-ray photon radiation, proton beams can be deposited in small, precise areas with minimal lateral scattering in tissue, ensuring that little to no radiation is delivered to healthy tissue surrounding the tumor^5^. This makes proton therapy the preferred option for treating central nervous malignancies in order to minimize neurocognitive deficits in normal brain tissue^6^,^7^,^8^. Recent studies show that proton radiation exerts significantly greater cytotoxic damage in the radiation-resistant, stem cell-like tumor cells in non-small cell lung cancer (NSCLC) than conventional photon radiation^9^. Interestingly, it is agreed that proton radiation only generates a 10% higher relative biological effectiveness (RBE) than photon radiation in most types of cells and tissue^10^,^11^. The effects of proton radiation on characterized cancer stem cells remain unclear, and understanding the cellular response and discovering the underlying mechanism for treatment-induced cytotoxicity will contribute to the optimization of radiation therapy and ultimately lead to the development of new therapeutic targets.

Here we sought to determine the efficacy of proton and photon therapies in patient-derived GSCs. Our data reveal that proton radiation induces greater DNA damage, cell cycle alteration, and cytotoxicity through reactive oxygen species (ROS).

## Results

### Proton radiation induces greater cytotoxicity in GSCs than photon radiation

**Figure 1:**
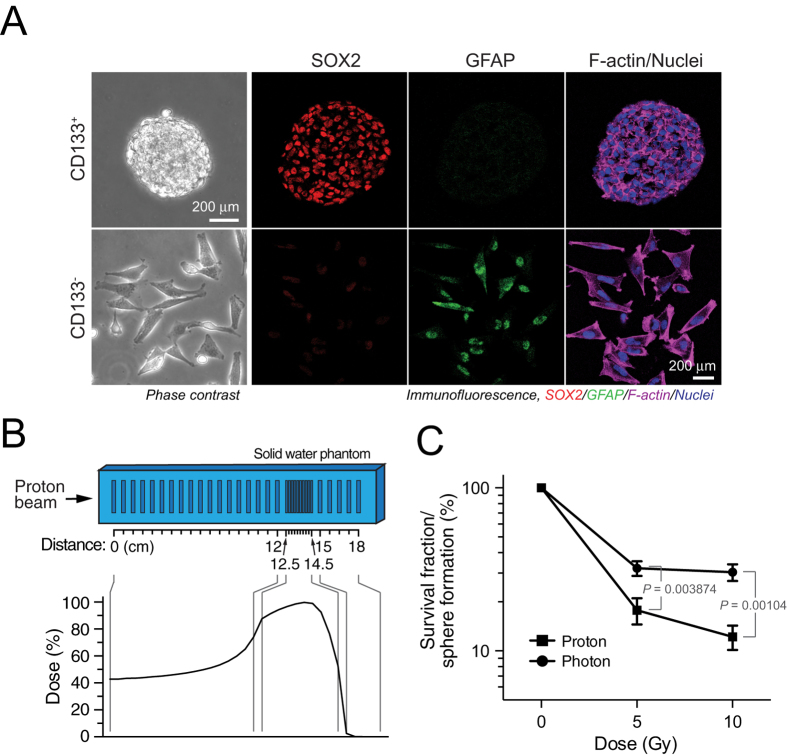
Proton radiation induces greater cytotoxicity in GSCs than photon radiation. (**A**) T4213 CD133^+^ and CD133^–^ cells were isolated from GBM patients, and stained with anti-SOX2 and anti-GFAP antibodies and Alexa Fluor 633-phalloidin. (**B**) Schematic of the solid water phantom used for proton irradiation. Shown are where cells were contained during irradiation and where the spread-out Bragg peak fell with regards to the phantom. (**C**) IN528 GSCs were irradiated and clonogenic survival fraction determined one week after radiation (means ± SEM, n = 18 from pooled four experiments, *p* values determined by a Student’s *t* test).

Patient-derived glioma stem cells (GSCs) have previously been isolated by flow cytometry and characterized in mouse tumor xenografts^4^. Consistent with their established stem cell specific activity^4^, T4213 CD133^+^ GSCs formed neurospheres *in vitro*, compared with adherent spreading cell morphology in CD133^–^ cells (Fig. 1A). Immunofluorescence analysis showed robust expression of the stem cell marker SOX2 and weak expression of the differentiation marker glial fibrillary acidic protein (GFAP) in CD133^+^ GSCs when compared to control CD133^–^ cells (Fig. 1A). Previous studies have shown that CD133^+^ GSCs exhibit robust resistance to photon radiation^4^. To investigate the effects of proton radiation on GSCs, we irradiated CD133^+^ cells with differing doses of proton beam radiation in a custom-made solid water phantom by utilizing the wells in the areas of interest: the proximal and distal edge of the spread-out Bragg peak (SOBP) as well as a group of wells proximal to the SOBP and large energy deposition (Fig. 1B). Our data show that proton radiation reduced the survival fraction of CD133^+^ cells more robustly than photon radiation, as indicated by more reduced sphere formation (Fig. 1C).

### Proton radiation induces stronger DNA damage in GSCs than photon radiation

**Figure 2:**
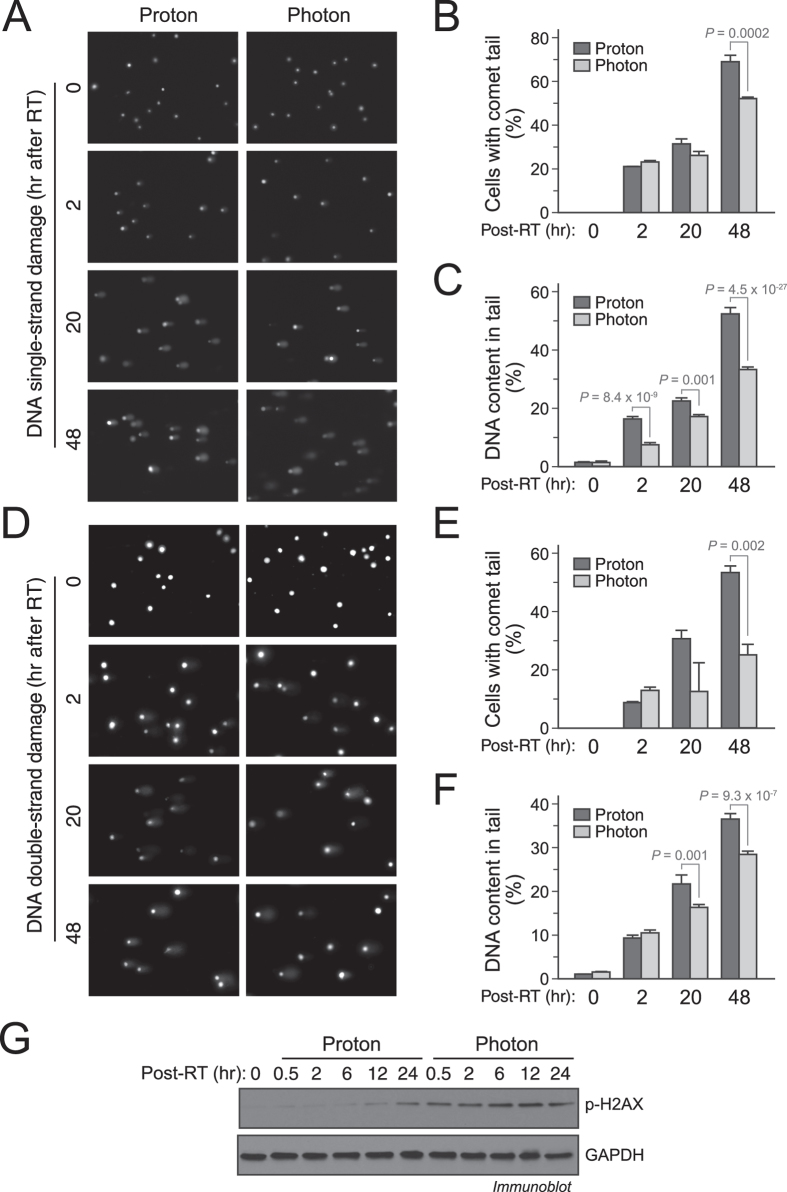
Proton radiation induces stronger DNA damage than photon radiation. IN528 GSCs were irradiated with 10 Gy of proton beam or x-ray photon radiation. (**A**–**C**) Post-irradiation DNA damage was assessed by single-cell gel electrophoresis assay under alkaline conditions (alkaline comet assay). (**A**) Representative images are shown. (**B**) Quantification of the percentages of cells with comet tails (means ± SD). (**C**) Quantification of the percentage of DNA in comet tails (means ± SD). (**D**–**F**) DNA damage was assessed by single-cell gel electrophoresis assay under neutral conditions (neutral comet assay). (**D**) Representative images are shown. (**E**) Quantification of the percentages of cells with comet tails (means ± SD). (**F**) Quantification of the percentage of DNA in comet tails (means ± SD). *p* values were determined by Student’s *t* tests. (**G**) Cell lysates were immunoblotted with anti-P-H2AX-Ser^139^ and anti-GAPDH antibodies. Full-length blots are presented in Supplementary Figure S4.

Radiation induces its major biological effects through DNA damage. Therefore, we examined the extent of DNA damage inflicted by each of the therapy modalities. Utilizing the DNA comet assay, we reveal that proton therapy produced significantly more cells containing single-stranded DNA breaks 48 hours after treatment (Fig. 2A,B) with more DNA content in each tail starting 2 hours after therapy (Fig. 2A,C). Similar results were observed with double-stranded DNA breaks (Fig. 2D–F). H2AX phosphorylation at Ser 139 is a hallmark for post-damage DNA repair^12^,^13^. Immunoblot analysis shows that photon radiation induced sustained H2AX phosphorylation (Fig. 2G), supporting the robust DNA repair ability of GSCs. In contrast, non-stem cells tend to exhibit decreasing H2AX phosphorylation over time with a peak phosphorylation detected around 0.5 to 2 hours post radiation. Interestingly, proton radiation induced a delayed H2AX phosphorylation compared to photon radiation (Fig. 2G), suggesting that the DNA repair is somehow inhibited by proton radiation. It is likely that proton radiation induces stronger DNA damage and inhibits DNA repair, leading to greater cytotoxicity in GSCs.

### Proton radiation alters Chk1/2 phosphorylation and shortens G2 arrest recovery

**Figure 3:**
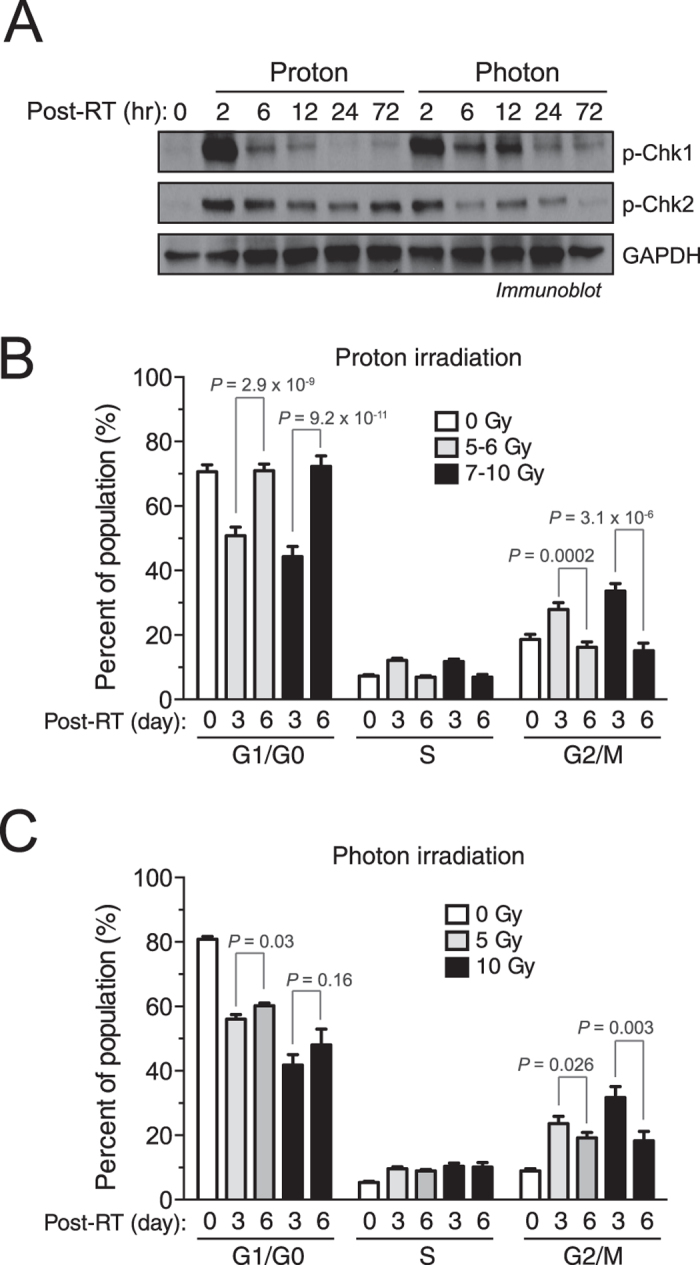
Proton radiation alters Chk1/2 phosphorylation and shortens G2 arrest recovery. IN528 GSCs were irradiated with 10 Gy of proton beam or x-ray photon radiation. (**A**) Cell lysates were immunoblotted with anti-P-Chk1-Ser^317^, anti-P-Chk2-Thr^68^, and anti-GAPDH antibodies. Full-length blots are presented in Supplementary Figure 1. (**B**,**C**) Cell Cycle was analyzed by flow cytometry (means ± SEM, n = 4–8). *p* values were determined by Student’s *t* tests. (**B**) Proton radiation (**C**) Photon radiation.

Checkpoint kinase (Chk) 1/2 is a master regulator of the cell cycle, and is critical for the initiation of cell cycle checkpoints and cell cycle arrest^14^. Chk1/2 plays an important role in DNA repair in GSCs^4^. Consistent with previous findings, our data show that photon radiation induced rapid phosphorylation of both Chk1 and Chk2 two hours after treatment, followed by a gradual decline (Fig. 3A). Importantly, proton radiation altered the pattern of Chk1/2 phosphorylation post treatment. It induced sustained Chk2 phosphorylation and more rapid Chk1 dephosphorylation (Fig. 3A). This may be responsible for the post-radiation redistribution of the cell cycle. Consistent with this hypothesis, proton radiation significantly reduced the recovery period of G2 and G0 arrest (from 3 to 6 days after irradiation), though a similar redistribution of G1/G0, S, and G2/M cell cycle was observed in the early phase (3 days after irradiation) in proton and photon treatments (Fig. 3B,C). Notably, G2 arrest, not G0 arrest, is critical for cell function regulation after radiation, and shortening the recovery period of G2 arrest results in less time for DNA repair, ultimately leading to more DNA damage^15^. Thus, proton radiation may evoke stronger Chk2 phosphorylation to reduce the recovery of G2 arrest, leading to more DNA damage. Furthermore, this may produce stronger cytotoxicity and cell apoptosis.

### Proton radiation induces stronger cell apoptosis than photon radiation

**Figure 4:**
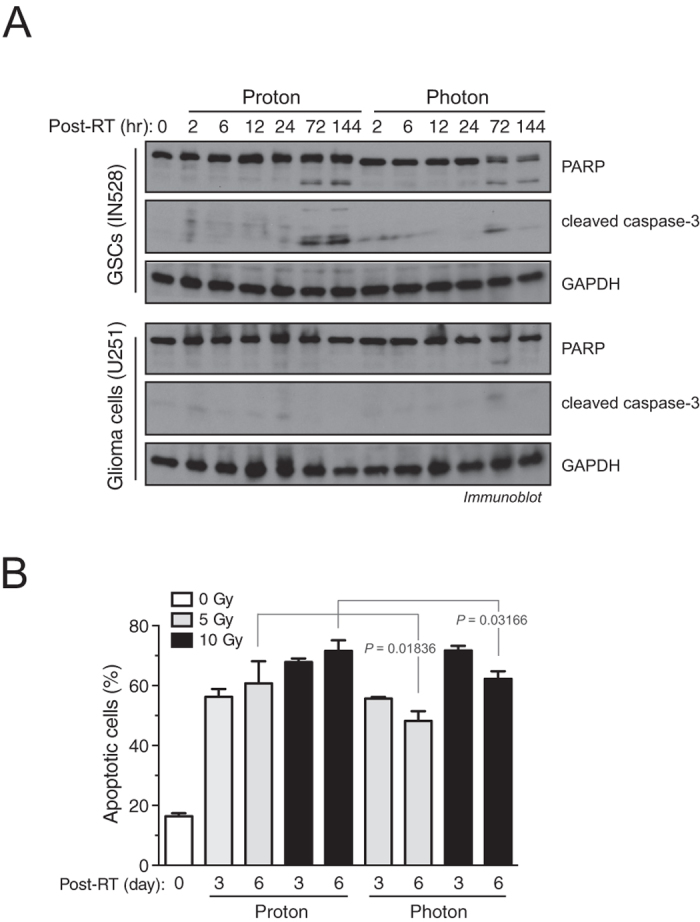
Proton radiation induces stronger cell apoptosis than photon radiation. IN528 GSCs and U251 glioma cells were irradiated with 10 Gy of proton beam or x-ray photon radiation. (**A**) Cell lysates were immunoblotted with anti-PARP, anti-cleaved caspase-3, and anti-GAPDH antibodies. Full-length blots are presented in Supplementary Figure 1. (**B**) Cell apoptosis was analyzed by flow cytometry (means ± SEM, n = 4–8). *p* values were determined by Student’s *t* tests.

To explore this possibility, we then determined the efficacy of both treatment modalities in inducing cell apoptosis. Central to cell apoptosis is caspase-3 and poly ADP-ribose polymerase (PARP) cleavage followed by activation and DNA fragmentation. Immunoblot analysis shows that proton radiation caused significantly stronger caspase-3 cleavage than photon radiation after irradiation in both IN528 (Fig. 4A) and T4213 GSCs (Supplementary Figure S2). Proton radiation also induced a stronger and more sustained PARP cleavage than photon radiation (Fig. 4A). Moreover, proton radiation did not induce more caspase-3 and PARP cleavage in U251 glioma cells (Fig. 4A). Consistent with these findings, proton radiation induced robust cell apoptosis in IN528 and T4213 GSCs but not in U251 glioma cells (Fig. 4B and Supplementary Figure S3), implicating a possible selectivity of proton therapy radiation-induced biological effectiveness toward GSCs. Interestingly, proton radiation-induced apoptosis continually increased over time (3–6 days), while photon radiation-induced apoptosis was reduced over the same time period (Fig. 4B). These findings suggest that proton radiation-induced DNA damage and cytotoxicity are more irreparable and lead to further cell apoptosis in GSCs when compared with photon therapy.

### Proton radiation induces robust generation of ROS to induce cytotoxicity

**Figure 5:**
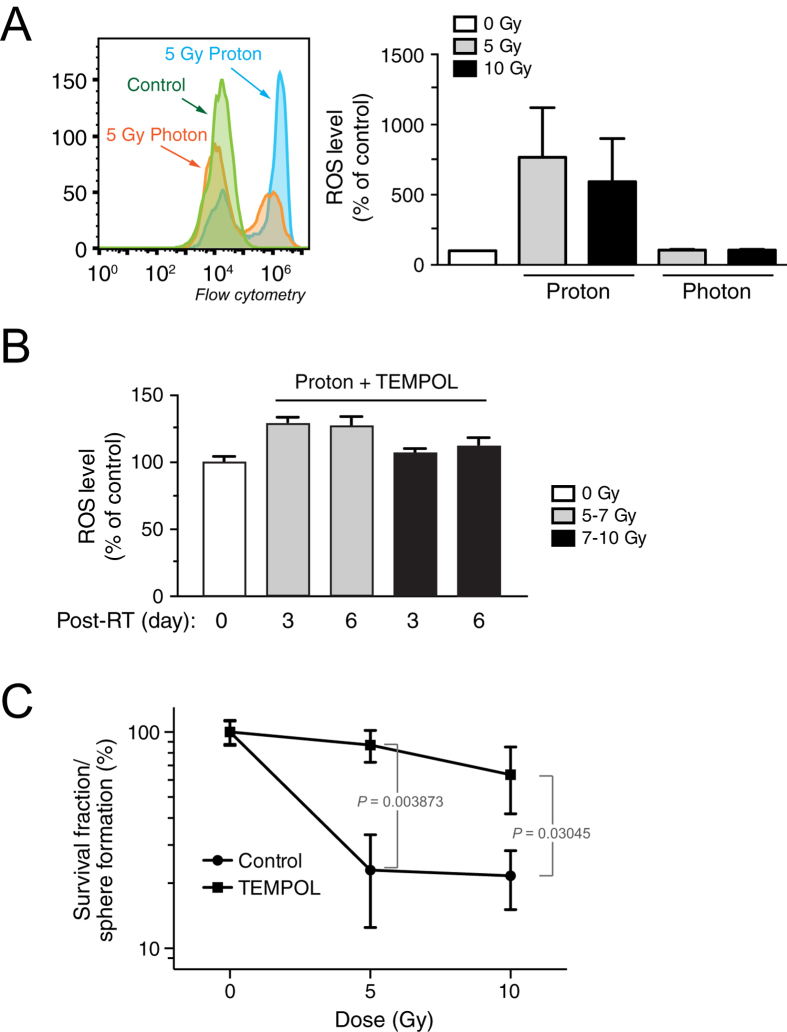
Proton radiation induces ROS-dependent cytotoxicity. IN528 GSCs were irradiated with proton beam or x-ray photon at different doses. (**A**) Intracellular ROS levels in GSCs were measured three days after irradiation by flow cytometry. Left, representative data. Right, Quantification of ROS level three days post irradiation (means ± SEM, n = 4–8). (**B**,**C**) Cells were treated with TEMPOL (10 mM) one hour prior to proton or photon irradiation. (**B**) ROS levels were determined by flow cytometry (means ± SEM, n = 9–10). (**C**) Cells were subjected to clonogenic survival analysis (means ± SEM, n = 5). *p* values were determined by Student’s *t* tests.

Radiation induces DNA damage and cytotoxicity through direct DNA breaks and indirectly through the generation of reactive oxygen species (ROS)^16^,^17^. Compared with photons, protons are charged particles with greater mass and have the ability to produce a higher ionization density region towards the distal edge of the SOBP, which will likely produce more ROS^5^. We explored the possible mechanism for proton radiation-induced biological effects by examining the role of ROS. Our data reveal that proton radiation induces a greater amount of ROS generation than photon radiation, with levels constantly increasing during the first 20 hours after radiation (Supplementary Figure S4) and enormous levels of intracellular ROS observed three days post irradiation, as indicated by the 7-fold increase (Fig. 5A). This suggests that ROS may play a critical role in the greater efficacy and associated cytotoxicity of proton therapy. To test this hypothesis, we treated GSCs with the ROS scavenger, 4-Hydroxy-Tempo (TEMPOL) (10 mM), one hour prior to therapy. This addition successfully abolished proton radiation-induced ROS generation (Fig. 5B) and our results indicate that pretreating GSCs with TEMPOL significantly prevented proton radiation-induced cytotoxicity, as evidenced by the almost complete restoration of clonogenic ability in 5 Gy proton beam-irradiated cells (Fig. 5C).

### ROS is required for proton radiation-induced DNA damage and repair

**Figure 6:**
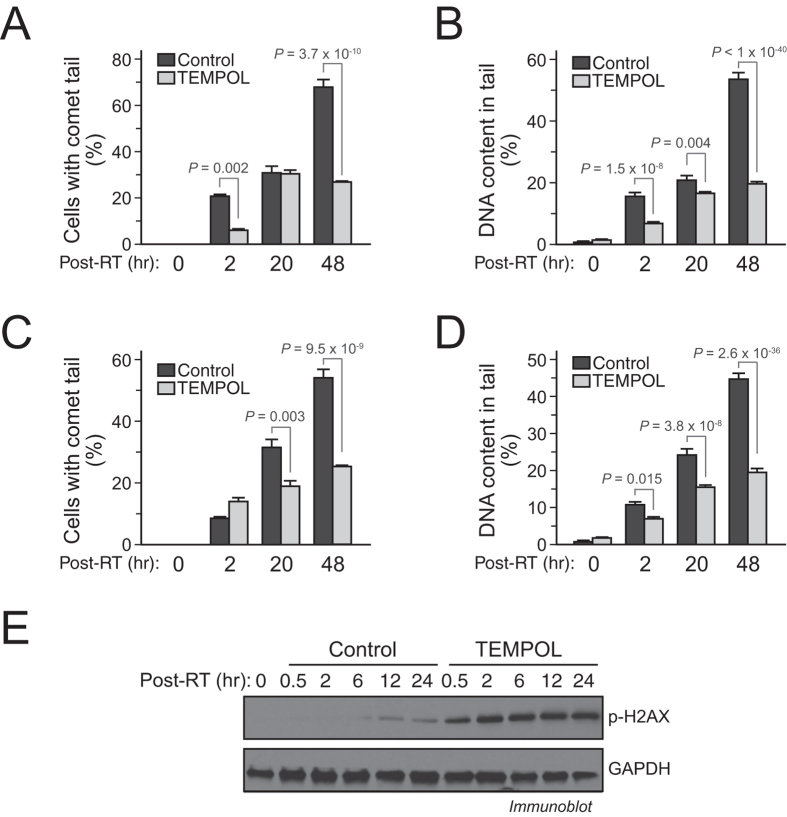
ROS is required for proton radiation-induced DNA damage and repair. IN528 GSCs were pre-treated with TEMPOL (10 mM) and irradiated with 10 Gy proton beam radiation. (**A**,**B**) Post-irradiation DNA damage was assessed by single-cell gel electrophoresis assay under alkaline conditions (alkaline comet assay). (**A**) Quantification of the percentages of cells with comet tails (means ± SD). (**B**) Quantification of the percentage of DNA in comet tails (means ± SD). (**C**,**D**) DNA damage was assessed by single-cell gel electrophoresis assay under neutral conditions (neutral comet assay). (**C**) Quantification of the percentages of cells with comet tails (means ± SD). (**D**) Quantification of the percentage of DNA in comet tails (means ± SD). (**E**) Cell lysates were immunoblotted with anti-P-H2AX-Ser^139^ and anti-GAPDH antibodies. *p* values were determined by Student’s *t* tests. Full-length blots are presented in Supplementary Figure S4.

We then investigated the role of ROS in proton radiation-induced DNA damage and repair. Our data show that pretreating cells with TEMPOL significantly reduced the extent of single-stranded and double-stranded DNA breaks (Fig. 6A–D), suggesting that proton radiation induces greater amounts of DNA damage through ROS generation. Moreover, incubation with TEMPOL more rapidly promoted H2AX phosphorylation, as shown by the increased and prolonged phosphorylation compared to proton therapy alone (Fig. 6E). This suggests a critical role for ROS in altering the repair ability of GSC after irradiation, and implicates that the large quantity of ROS produced by proton therapy plays a major role in its cytotoxicity.

### ROS is required for proton radiation-induced cell cycle redistribution and apoptosis

**Figure 7:**
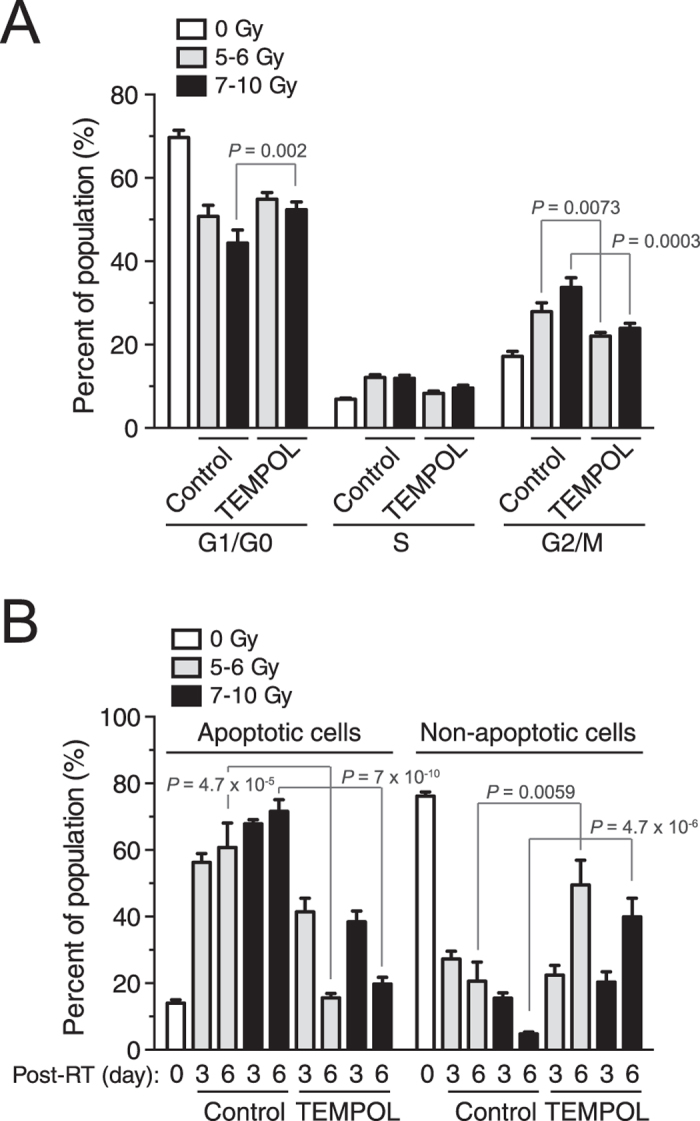
ROS is required for proton radiation-induced cell cycle redistribution and apoptosis. IN528 GSCs were pre-treated with TEMPOL (10 mM) one hour before irradiation and irradiated with at different doses using the double scattering modality of proton radiation. (**A**) Cell Cycle was analyzed by flow cytometry (means ± SEM, n = 5–10). (**B**) Cell apoptosis was analyzed by flow cytometry (means ± SEM, n = 5–10). *p* values were determined by Student’s *t* tests.

Finally, we determined the role of ROS in the proton radiation-induced redistribution of the cell cycle and induction of apoptosis. Our data show that TEMPOL treatment significantly inhibited the G2 arrest in GSCs (Fig. 7A). This allowed the DNA damage to be repaired and produced significantly diminished cell apoptosis 3 days post therapy (Fig. 7B). Additionally, there was remarkably improved cell recovery, as indicated by the dramatic increase in the population of healthy, non-apoptotic cells and the decrease in the population of apoptotic cells (Fig. 7B). These findings further suggest that proton radiation induces cell cycle redistribution and apoptosis in a ROS-dependent fashion.

## Discussion

Our study shows that proton radiation induces more robust DNA damage, cytotoxicity, and cell apoptosis than photon radiation, for the first time when using characterized cancer stem cells. We reveal that this enhanced efficacy is associated with increased DNA damage, reduced DNA repair, altered cell cycle distribution, and increased caspase-3 activity. Furthermore, the fact that the introduction of the ROS scavenger TEMPOL lessened the effects of proton therapy suggests a ROS-dependent mechanism.

Cancer stem cells have been well characterized in GBM^3^,^4^. Previous studies have demonstrated a critical role of GSCs in the radioresistance of GBM^4^. Therefore, targeting and eliminating GSCs is critical for overcoming the therapeutic resistance in GBM^18^,^19^,^20^. Although most identified GSCs are radioresistant, some GSCs show a range of radiosensitivities^21^,^22^, possibly due to different isolation and culture approaches, population heterogeneity, and different genetic statuses (proneural, neural, classical, or mesenchymal). Most cancer stem cells are highly refractory to conventional radiation and chemotherapy, largely due to their intrinsic abilities to repair DNA and scavenge free radicals^23^,^24^, e.g., breast cancer stem cells have increased ROS mediation and enhanced DNA repair. However, these characteristics have not been well established in GSCs. Here, our data suggest that the generation of ROS is critical for DNA damage and diminished repair in GSCs. Our study consistently shows that x-ray photon radiation induces minimal amounts of DNA damage and produces smaller quantities of ROS in GSCs, providing an explanation for its therapeutic ineffectiveness. More importantly, our data indicate that proton radiation evokes marked ROS generation, which likely exceeds the tolerable ROS threshold and the scavenging ability of GSCs, and ultimately leads to cell apoptosis. Likewise, recent studies show that proton radiation induces ROS production in multiple types of cancer cells^25^. In addition, the delayed H2AX phosphorylation induced by proton radiation in GSCs suggests that proton radiation also inhibits DNA repair, possibly because the high levels of ROS generated may alter the activity levels of key DNA repair kinases including ATM, ATR, and DNA-PK.

As positively charged particles, protons cause DNA damage and cytotoxicity by direct collisions with DNA molecules and by indirect DNA damage through the generation of ROS and are more efficient than photons due to their relatively high beam energies used for radiotherapy (typically 70 to 250 MeV, compared with 4 to 20 MeV for x-ray radiation)^5^. However, here we show that the major cytotoxic effect of proton radiation on GSCs is through the generation of large quantities of intracellular ROS and its indirect effects as made evident by the diminished DNA damage and cell apoptosis upon the introduction of the ROS scavenger TEMPOL prior to treatment. The robust ROS generation associated with proton radiation may be due to its direct effects and/or subsequent radiation-induced mitochondrial dysfunction. Consistent with this idea, pretreatment with radical scavengers can increase cell survival after radiation^26^.

Given that proton radiation induces only a 10% higher relative biological effectiveness (RBE) in most types of cells and tissues when compared to conventional photon therapy, it has been generally accepted that proton therapy is unlikely to improve overall patient survival^10^,^11^. However, recent results show that proton therapy provides better local control in NSCLC and meningioma^27^,^28^,^29^. In fact, the estimates of RBE depend on the cell type and the chosen detection methods because it has been shown that DNA damage and apoptotic responses vary greatly between gamma radiation and proton therapy in a tissue- and dose-dependent fashion^30^. Interestingly, previous data indicate that proton radiation induces more cell apoptosis than photon radiation in various cancer cell types^31^. Furthermore, a recent study shows that proton radiation has selective cytotoxic effects on lung cancer stem cell-like cells^9^. We hypothesize that it is due to the fact that intrinsic ROS tolerance and ROS generation vary in different cell types and treatment modalities. In addition, proton beam therapy has a higher linear energy transfer (LET) rate, particularly toward the distal edge of the SOBP, compared with conventional X-ray photon radiation. Higher LET may enhance RBE, which is supported by an increasing RBE with proton treatment depth^11^. However, the specific role of LET in radiation effects remains largely unknown.

In summary, we show that proton radiation eradicates GSCs more efficiently than photon radiation, through the production of ROS. These findings suggest proton radiation as the preferred therapy for treating GBM, particularly for its ability to eliminate GSCs. Dynamic biological effects observed in GSCs post-treatment including cell cycle redistribution, DNA damage and repair, and ROS generation offer some insight into the cellular response of GSCs to proton radiation. More importantly, this study implicates that with optimized dose fractionation proton therapy could ultimately improve the clinical outcome of GBM patients.

## Methods

### Cell Culture

Patient-derived glioma stem cells (GSCs), IN528 and T4213, were kindly provided by Dr. Jeremy Rich (Cleveland Clinic). GSCs were cultured in serum-free Neurobasal-A medium (Gibco), supplemented with B-27 Supplement Minus Vitamin A (Gibco), GlutaMax (Gibco), sodium pyruvate (Gibco), fibroblastic growth factor (5 ng/ml, R&D Systems), and epidermal growth factor (20 ng/ml, R&D Systems) at 37 °C in a humidified atmosphere of air containing 5% CO2. Cells were maintained and limited to a low number of passages (less than five) from their removal from cryopreservation before each set was exposed to radiation. Human U251 glioma cells (ATCC) were cultured in DMEM medium (Gibco) supplemented with 5% fetal bovine serum (FBS, Gibco).

### Photon and Proton Irradiation

For photon radiation, cells were added to 6-well plates and treated with 0, 5, or 10 Gy of x-ray radiation at a dose rate of 2 Gy/min using the X-Rad 320ix cabinet system (Precision X-Ray). For proton therapy, single cell suspensions of GSCs were prepared by using TrypLE (Gibco), and cultured in non-tissue-culture-treated plates for one day prior to irradiation. GSCs were then loaded into the wells of a custom solid water phantom with a size of 20 × 5 × 2 cm (Length, width, and height). There are three regions of interest: one group of wells prior to the large energy deposition, and groups of wells near the proximal and distal edge of the spread-out Bragg peak (SOBP) (Fig. 1B), and the dose that each well (30 × 15 × 2 mm or 30 × 15 × 1 mm, length, width, and height) received was calculated and recorded. U251 cells were cultured in tissue-culture-treated flasks. Cells were treated with 0, 5, or 10 Gy of double scattering proton irradiation at a dose rate of 2 Gy/min at the Roberts Proton Therapy Center in our department.

### Clonogenic/Sphere Formation Assay

Irradiated IN528 cells were seeded into six-well non-tissue-culture-treated plates at a density of 2,000 cells/well, and cultured for 7 days. The numbers of formed spheres were counted at 7 days post-irradiation.

### Comet Assay

Irradiated IN528 cells were subjected to alkaline and neutral comet assays (single-cell gel electrophoresis) for detecting DNA single- and double- strand breaks, respectively, as previously described^32^. In brief, irradiated cells were washed with PBS and mixed with low melting agarose (1:10), before being loaded onto microscope slides. Cell lysis was performed at 4 °C (alkaline comet assay) or 37 °C (neutral comet assays). After electrophoresis for 25 minutes at room temperature, DNA was stained with propidium iodide and imaged with an AxioImager A1 fluorescence microscope (Zeiss) equipped with an AxioCam 506 CCD camera (Zeiss). The cell number with DNA comets and the DNA percent content in comet tail region were measured using ImageJ and OpenComet 1.3 software (three assays, each with about 200 cells analyzed).

### Immunofluorescence

Neurospheres of T4213 CD133^+^ cells were collected by centrifugation at 200 × *g* for 10 minutes and washed with PBS, followed by immersion in Tissue-Tek OCT compound (Sakura). Frozen sections were prepared. T4213 CD133^–^ cells on culture slides were washed with PBS and fixed with 3% paraformaldehyde. The sections and culture slides were stained with anti-SOX2 (1:200, Abcam) and anti-GFAP (1:100, R&D System) antibodies, and visualized by using Alexa Fluor 488- and 568- conjugated IgGs and Alexa Fluor 633-labeled phalloidin (Invitrogen). Cells were examined by confocal scanning using a TCS-SP microscope (Leica, Heidelberg, Germany).

### Immunoblot

Cells were lysed using cell lysis buffer (Cell Signaling) with complete protease inhibitor cocktail (1:1,000, Roche) and Halt phosphatase inhibitor cocktail (1:100, Thermo). After total protein quantification using a Bradford reagent (Bio-Rad), 20 μg of cell lysate protein was resolved by 4–15% SDS-PAGE (Bio-Rad) under denaturing conditions, and transferred to PVDF membranes. Membranes were blocked in 5% BSA and then incubated with anti- phospho-H2AX (1:1,000, Cell Signaling), phospho-Chk1-Ser^317^ (1:1,000; Cell Signaling), phospho-Chk2-Thr^68^ (1:1,000, Cell Signaling), caspase-3 (1:1,000; Cell Signaling), cleaved caspse-3 (1:1,000; Cell Signaling), PARP (1:1,000; Cell Signaling), and GAPDH (1:5,000, Cell Signaling) antibodies, followed by incubation with a secondary antibody conjugated with horseradish peroxidase (HRP) (1:2,000; Cell Signaling). Signals were visualized using ECL Prime Western Blotting Detection reagent (Amersham).

### Cell Cycle Analysis

Irradiated IN528 cells were washed with Hank’s Balanced Salt Solution (HBSS), fixed with 4% paraformaldehyde for 15 minutes, and then permeabilized with 0.1% Triton X-100. Cells were stained with propidium iodide (20 μg/ml, BD Pharmingen) in the presence of DNase-free RNase A (0.2 mg/ml, Thermo). 1 × 10^6^ Cells were analyzed with an Accuri C6 flow cytometer (BD Biosciences), and data analyzed using FlowJo software.

### Cell Apoptosis Assay

Cells were washed with PBS and stained with FITC-conjugated annexin V and propidium iodide (100 μg/ml, BD Pharmingen), using an Apoptosis Detection Kit I (BD Pharmingen). 1 × 10^6^ Cells were analyzed with an Accuri C6 flow cytometer (BD Biosciences), and data analyzed using FlowJo software.

### Reactive Oxygen Species Assay

Irradiated IN528 cells were washed with HBSS and incubated with the ROS detection reagent utilizing a Total Reactive Oxygen Species Assay Kit (eBioscience), according to the manufacturer’s instruction. For ROS scavenging assays, IN528 cells were incubated with 10 mM 4-Hydroxy-TEMPO (TEMPOL, Sigma) one hour prior to proton irradiation.

### Statistical Analysis

Student’s *t* and ANOVA tests were used for statistical analysis between groups using GraphPad Prism 6 and KaleidaGraph software, and *p* values less than 0.05 were considered to represent a statistically significant difference.

## Additional Information

**How to cite this article**: Alan Mitteer, R. *et al.* Proton beam radiation induces DNA damage and cell apoptosis in glioma stem cells through reactive oxygen species. *Sci. Rep.* **5**, 13961; doi: 10.1038/srep13961 (2015).

## Supplementary Material

Supplementary Figures
