# Supplementary material for: Proton beam radiation induces DNA damage and cell apoptosis in glioma stem cells through reactive oxygen species
Source: Sci Rep. 2015 Sep 10;5:13961. doi: 10.1038/srep13961 (PMC4564801; doi:10.1038/srep13961)
Supplement: Supplementary Figures [file srep13961-s1.pdf]

Proton beam radiation induces DNA damage and cell apoptosis in glioma stem cells through reactive oxygen species

R. Alan Mitteer Jr.[1,*], Yanling Wang[1,*], Jennifer Shah[1,2], Sherika Gordon[1,3], Marcus Fager[1], Param-Puneet Butter[1,3], Hyun Jun Kim[1], Consuelo Guardiola-Salmeron[1], Alejandro Carabe-Fernandez[1] & Yi Fan[1]

[1]Department of Radiation Oncology, University of Pennsylvania Perelman School of Medicine, Philadelphia, Pennsylvania, USA 19104

[2]Lehigh University, Bethlehem, Pennsylvania, USA 18015

[3]Drexel University, Philadelphia, Pennsylvania, USA 19104

[*]Co-first author

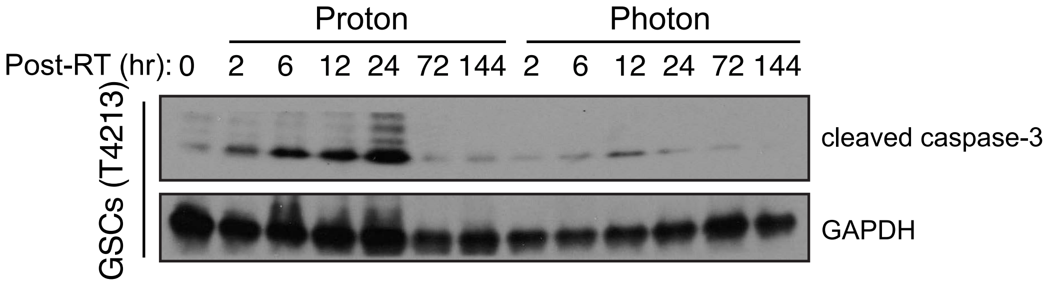

Supplementary Figure S1. Proton radiation induces caspase-3 cleavage in GSCs.
T4213 GSCs were irradiated with 10 Gy of proton beam or x-ray photon radiation. Cell lysates were immunoblotted with anti-cleaved caspase-3 and anti-GAPDH antibodies.

A

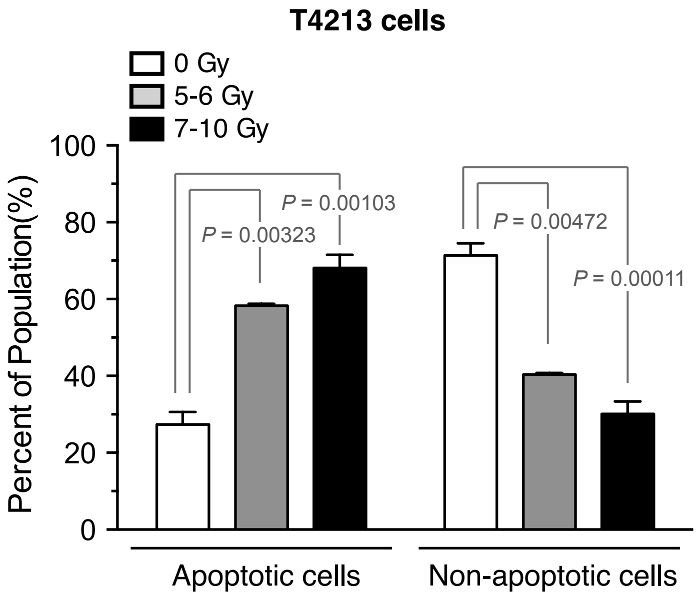

B

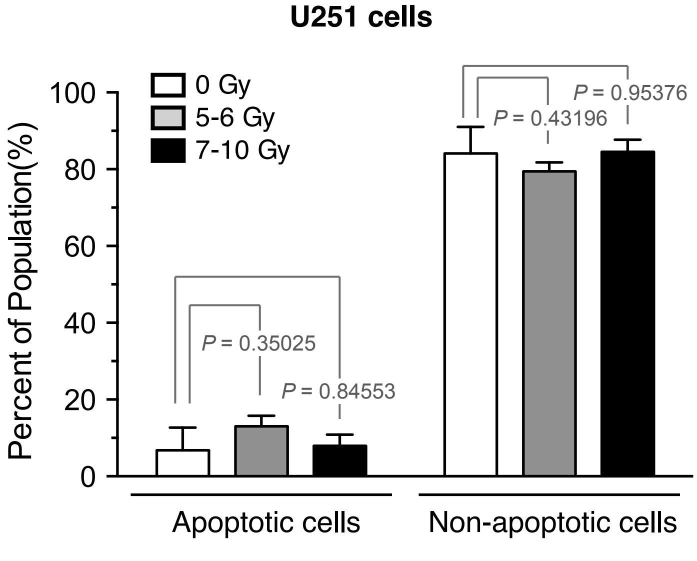

Supplementary Figure S2. Proton radiation induces cell apoptosis in T4213 GSCs but not in U251 glioma cells.
T4213 GSCs and U251 glioma cells were irradiated by proton beam. Three days after irradiation, cells were stained FITC-conjugated annexin V and propidium Iodide, followed by flow cytometry analysis for apoptosis (means ± SEM, n = 5-8, *p* values determined by ANOVA test).

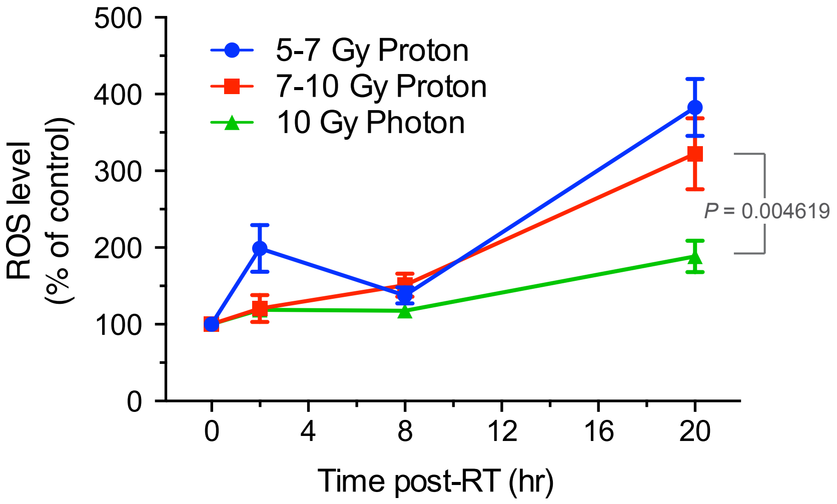

Supplementary Figure S3. Proton radiation induces robust ROS production in GSCs.
IN528 GSCs were irradiated with proton beam or photon. Intracellular ROS levels were determined by flow cytometry analysis (means ± SEM, n = 6-8, *p* values determined by Student *t* test).

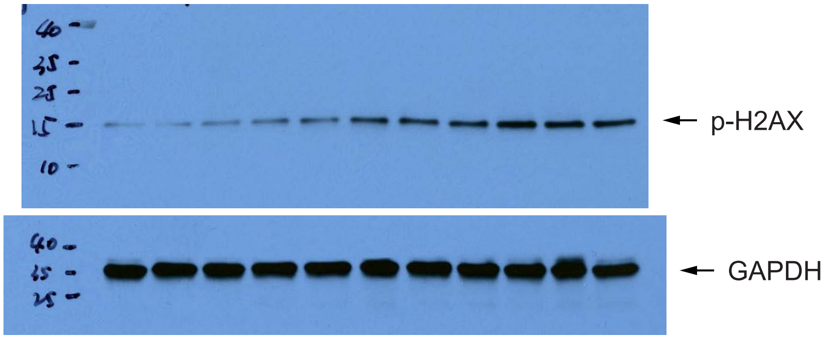

Figure 2G

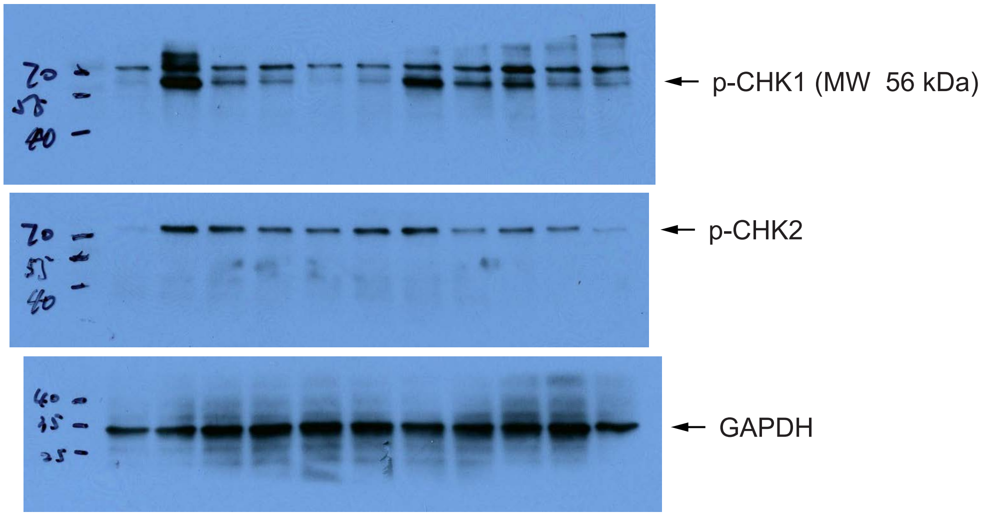

Figure 3A

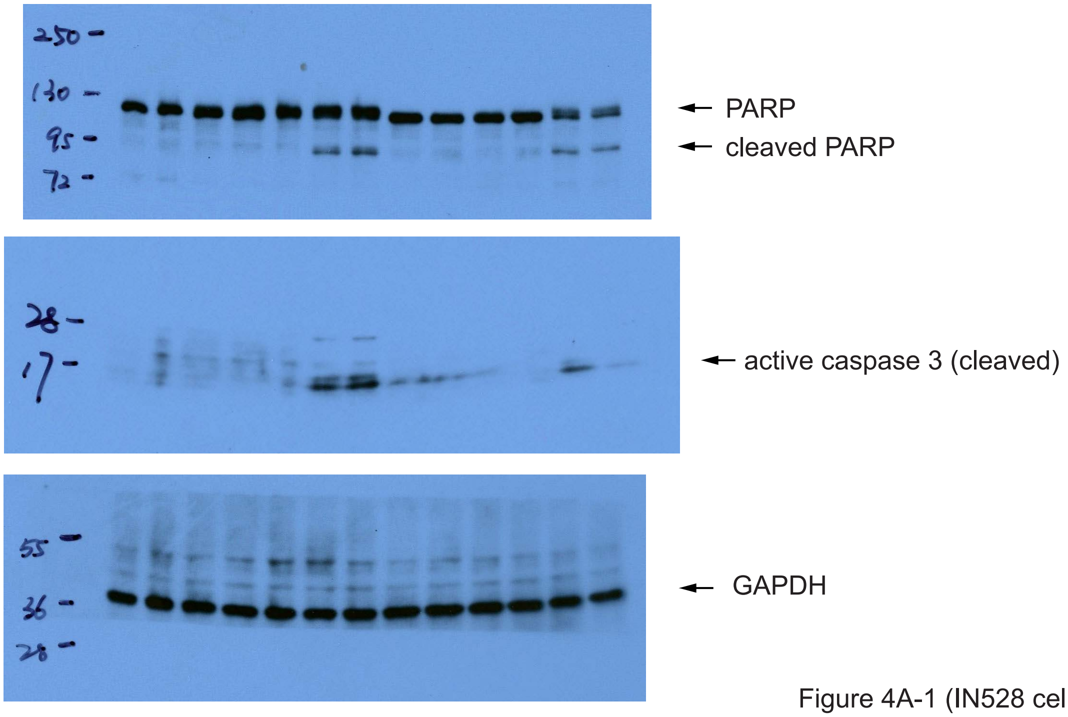

Figure 4A-1 (IN528 cells)

Supplementary Figure S4. Full-length blots.

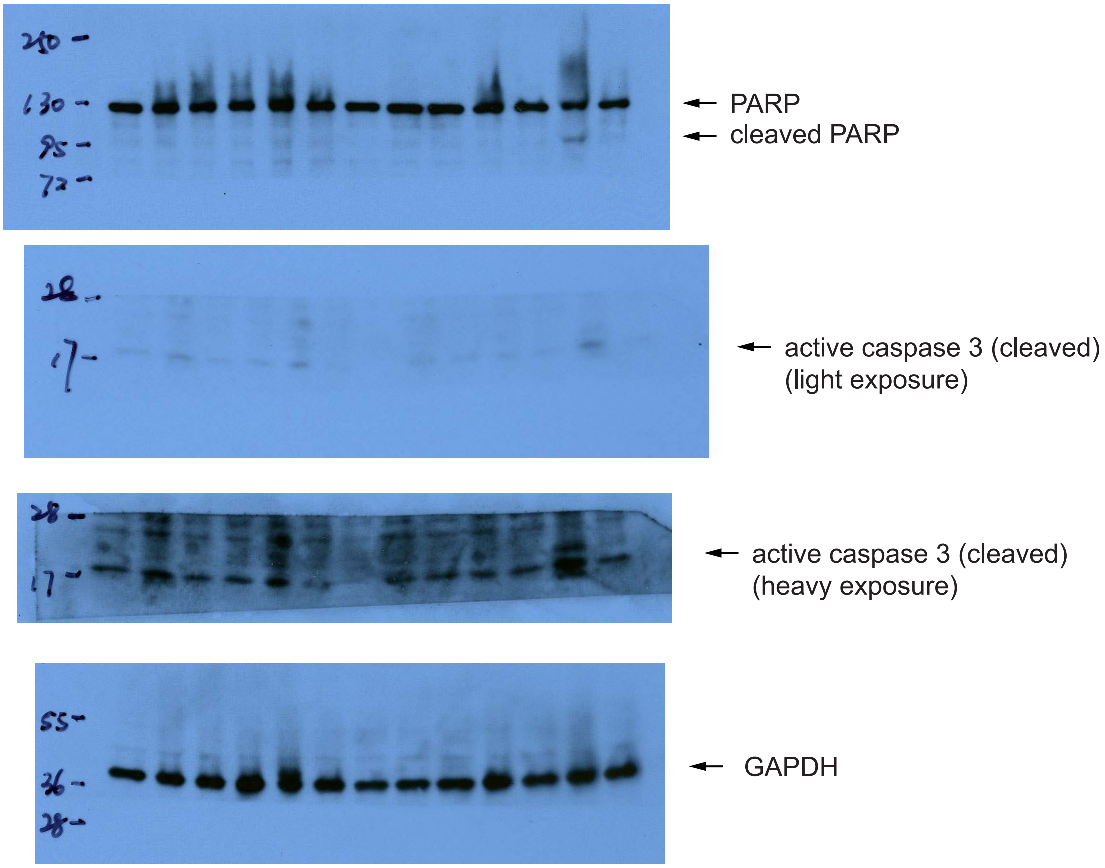

Figure 4A-2 (U251 cells)

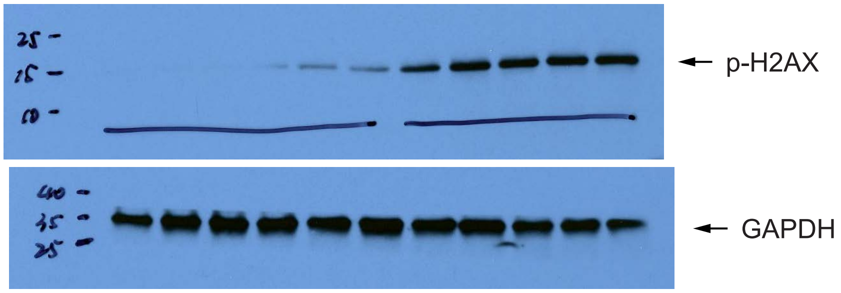

Figure 6E

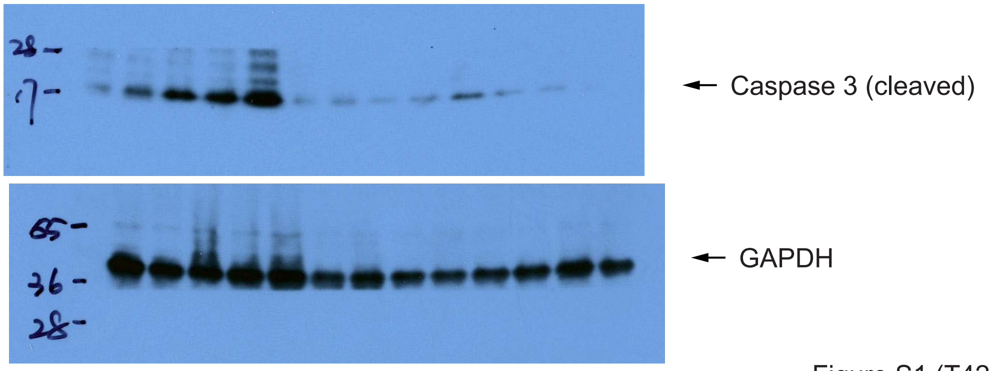

Figure S1 (T4213 cells)

Supplementary Figure S4. Full-length blots (continued).
